# Quercetin alleviates ulcerative colitis through inhibiting CXCL8-CXCR1/2 axis: a network and transcriptome analysis

**DOI:** 10.3389/fphar.2024.1485255

**Published:** 2024-12-09

**Authors:** Zhangyu Jiang, Mingjuan Yan, Yanmi Qin, Zhenglin Liu, Yilin Duan, Yingju Wang, Ruisen Zhang, Wenjia Lin, Yanwu Li, Tian Xie, Junyu Ke

**Affiliations:** ^1^ International Institute for Translational Chinese Medicine, Guangzhou University of Chinese Medicine, Guangzhou, China; ^2^ Department of Pharmacy, Guangzhou University of Chinese Medicine, Guangzhou, China; ^3^ Department of Cardiology, Shenzhen Hospital (Futian) of Guangzhou University of Chinese Medicine, Shenzhen, China; ^4^ The First Affiliated Hospital of Guangzhou University of Chinese Medicine, Guangzhou, China; ^5^ School of Basic Medical Sciences, Guangzhou University of Chinese Medicine, Guangzhou, China; ^6^ Foshan Chancheng Center Hospital of Guangzhou University of Chinese Medicine, Foshan, China; ^7^ Science and Technology Innovation Center, Guangzhou University of Chinese Medicine, Guangzhou, China; ^8^ Gaozhou Hospital of Traditional Chinese Medicine Affiliated to Guangzhou University of Chinese Medicine, Gaozhou, China

**Keywords:** ulcerative colitis, quercetin, network analysis, intestinal mucosal barrier, transcriptome, CXCL8-CXCR1/2 axis

## Abstract

**Introduction:**

Ulcerative colitis (UC) is a chronic inflammatory condition of the intestinal tract in which mucosal healing is a crucial measure of therapeutic efficacy. Quercetin, a flavonoid prevalent in various foods and traditional Chinese medicines, exhibits notable pharmacological properties, including antioxidant and anti-inflammatory activities. Consequently, it warrants investigation to determine its potential therapeutic effects on UC. The objective of this study was to investigate the effects and underlying mechanisms of quercetin in a murine model of UC.

**Methods:**

A comprehensive approach integrating network predictions with transcriptomic analyses was employed to identify the potential targets and enriched pathways associated with quercetin in UC. Subsequently, the effects of quercetin on pathological morphology, inflammatory mediators, and mucosal barrier-associated proteins, as well as the identified potential targets and enriched pathways, were systematically investigated in a murine model of dextran sulfate sodium (DSS)-induced UC.

**Results:**

Network analyses identified CXCL8 and its receptors, CXCR1 and CXCR2, as primary target genes for therapeutic intervention in UC. Further validation through transcriptomic analysis and immunofluorescence staining demonstrated significant upregulation of the CXCL8-CXCR1/2 axis in the intestinal tissues of patients with UC. Experimental investigations in animal models have shown that quercetin markedly alleviates DSS-induced symptoms in mice. This effect includes the restoration of colonic crypt architecture, normalization of goblet cell structure and density, reduction of inflammatory cell infiltration, and decreased concentrations of inflammatory mediators. Quercetin enhanced the expression of tight junction (TJ) proteins, including ZO-1, MUC2 (Mucin 2), and occludin, thereby preserving the integrity of the intestinal mucosal barrier. Additionally, it significantly diminished the levels of IL-17A, NF-κB, CXCL8, CXCR1, and CXCR2 in the colonic tissues of mice with UC.

**Discussion:**

The ameliorative effects of quercetin on colon tissue damage in DSS-induced UC mice were significant, possibly due to its ability to inhibit the CXCL8-CXCR1/2 signaling axis. These findings provide a solid foundation for the clinical application and pharmaceutical advancement of quercetin.

## 1 Introduction

Ulcerative colitis (UC) is a chronic inflammatory condition characterized by widespread inflammation, challenging treatments options, prolonged disease duration, and frequent relapses (Le Berre et al., 2023). The etiology of UC remains unclear, although current understanding suggests an association with genetic factors, environmental influences, disturbances in intestinal microecology, compromised intestinal mucosal barriers, psychosocial aspects, and immune system dysregulation, among others (Meisman et al., 2020). The primary medications used for treating UC include glucocorticoids, immunosuppressants, and targeted therapies; however, their long-term use can lead to complications (Horio et al., 2024), drug resistance, and hormone dependence (Sit et al., 2019). This significantly impacts patients’ quality of life, underscoring the urgent need for safer and more effective alternative therapies.

Inflammation of the intestinal mucosa and immune factors are critical in the pathogenesis of UC (Chen et al., 2018; Gao et al., 2023). Current research indicates that CXCR1 and CXCR2 are receptors for CXCL8 and play significant roles in non-immune cells. Their functions involve various physiological and pathological processes, including the regulation of inflammatory responses, such as the release of inflammatory mediators and migration of inflammatory cells. When foreign pathogens invade cells, they trigger a localized release of inflammatory factors. The CXCL8-CXCR1/2 signaling pathway promotes neutrophil migration to inflamed or injured tissues, thereby enhancing inflammation. This pathway is vital in immune-inflammatory diseases (Sitaru et al., 2023a; Zhang et al., 2023; Zhu et al., 2021). Although the biological functions of CXCL8 and its receptors, CXCR1 and CXCR2, have been extensively studied, their expression and role in patients with UC remain controversial. Therefore, identifying candidate drugs that target the CXCL8-CXCR1/2 axis may represent a potential anti-inflammatory strategy for UC treatment.

Herbal monomers provide key chemical diversity, facilitating the search for active molecules that target the CXCL8-CXCR1/2 axis. Quercetin (Que), a flavonoid found in many plants, exhibits antioxidant, anti-inflammatory, antimicrobial, antitumor, and antifibrotic benefits (Qi et al., 2022; Tang et al., 2020). No articles have been published on the treatment of UC with Que through the CXCL8-CXCR1/2 signaling pathway. Furthermore, several herbal medicines (e.g., Baihua snake tongue grass, Xianhe Cao, Fishwort) have demonstrated efficacy with the key target of these herbal medicines being the CXCL8-CXCR1/2 axis (Li et al., 2020; Li et al., 2021; Wang et al., 2018). The results of this study suggest that Que may have therapeutic effects in UC. To our knowledge, this is the first investigation of Que in this context. Que significantly protects against mucosal inflammation and barrier dysfunction induced by dextran sulfate sodium (DSS). Que inhibits the CXCL8-CXCR1/2 axis, thereby improving UC by reducing intestinal inflammation and restoring barrier integrity.

## 2 Materials and methods

### 2.1 Network pharmacology analysis

#### 2.1.1 Access to Que targets

Que targets were identified in TCMSP(Ru et al., 2014) (https://old.tcmsp-e.com/tcmsp.php) and Swiss Target Prediction System (Gfeller et al., 2013) (http://www.example.compper/). Duplicate entries were removed, and the remaining targets were imported into the UniProt database to standardize their names and obtain drug-related targets (Chen et al., 2017).

#### 2.1.2 Identification of disease-associated targets

The Gene Cards (Safran et al., 2010) (https://www.genecards.org/), OMIM (Amberger et al., 2015) (www.omim.org/), and DisGeNET (https://www.disgenet.org/home/) databases were searched using the keywords “ulcerative colitis.” Following this, the final UC disease targets were standardised in the Uniprot database.

#### 2.1.3 Constructing the PPI network

Que-related and UC diseases were identified using the Venny 2.1 online mapping tool (https://bioinfogp.cnb.csic.es/tools/venny/index.html). The STRING v. 11.5 database (Szklarczyk et al., 2019) (http: cn.string-db.org) was restricted to “*Homo sapiens*” species and had a critical confidence score of >0.9. Cytoscape software (version 3.9.1) was used for building and visualizing the PPI network (Shannon et al., 2003).

#### 2.1.4 GO and KEGG enrichment analyses

Utilizing the GO (http://geneontology.org) and KEGG (http://www.genome.jp/kegg/) databases, functional enrichment and pathway enrichment analyses were performed (Zhou et al., 2019). For the analysis, we used KEGG pathway analysis and selected the species as “*H. sapiens*” (*p* ≤ 0.05).

### 2.2 CXCL8/CXCR1/2 protein expression: multiple fluorescent immunohistochemical staining

Participants who underwent colonoscopies between August 2021 and October 2025 provided samples for this study with informed consent and Ethics Committee approval from the Gaozhou Hospital of Traditional Chinese Medicine. Human subjects were included in this study, in compliance with the Declaration of Helsinki by the World Medical Association. Specimens from patients with ulcerative nodulitis were fixed in formalin within 10 min of excision, prior to paraffin embedding. The remaining specimens were frozen at −80°C for future use. (Ethical approval: yxllyjky20210812S).

Sections were incubated in an oven at 60°C for 1 h. Sections were then incubated overnight at 4°C with the primary antibodies CXCL8/CXCR1/2 at a dilution of 1:600, in accordance with the manufacturer’s instructions (Absin). Subsequently, the sections were stained, and the staining pattern was verified using fluorescence microscopy at a staining concentration of 1:100. Finally, the samples were counterstained with DAPI (1:100 dilution) and mounted using an anti-fade medium. Each case was imaged with an Olympus slide scanner and analyzed by a pathologist using the ImageJ software.

Participants who underwent colonoscopies between August 2021 and October 2025 provided samples for this research, with informed consent and Ethics Committee approval from Gaozhou Hospital of Traditional Chinese Medicine. Human subjects were used in these studies in compliance with the World Medical Association’s Declaration of Helsinki. Specimens from patients with ulcerative nodulitis were fixed in formalin within 10 min immediately after excision, prior to paraffin embedding. The remaining specimens were frozen at −80°C for future use. (Ethical number: yxllyjky20210812).

Initially, the sections were incubated in an oven at 60°C for 1 h. Following this, the sections were incubated overnight at 4°C with the primary antibody CXCL8/CXCR1/2 at a dilution of 1:600, in accordance with the manufacturer’s instructions (Absin). Subsequently, the sections were stained, and the staining pattern was verified using fluorescence microscopy at a staining concentration of 1:100. Finally, the samples were counterstained with DAPI at a 1:100 dilution and mounted using an anti-fade medium. Each case was imaged with an Olympus slide scanner and analyzed by a pathologist using ImageJ software.

### 2.3 Transcriptome analysis

#### 2.3.1 Transcriptome data acquisition

We combined two GEO databases to complete the transcriptome study, including GSE59071 (Vanhove et al., 2015) and GSE206171 (Soendergaard et al., 2017). This study included 74 active UC and 11 control samples from GSE59071 and 114 UC and 38 control samples from GSE206171.

#### 2.3.2 Data quality control and preprocessing

We conducted the analysis using R (version 4.0.2). Initially, the raw microarray data were background-corrected, quantile normalized, and standardized using the Robust Multichip Average algorithm from the R package “affy.” The probe IDs from the microarray were then mapped to gene names using the annotation package “hugene10sttranscriptcluster.db” based on the GPL6244 platform and the package “hug219.db” based on the GPL13667 platform, respectively.

Batch effects needed to be addressed due to differences between databases. We used the function removeBatchEffect in the package “limma” to eliminate batch effects from the two different data platforms.

Subsequently, principal component analysis was performed on the gene counts, and visualizations were generated to assess the biological reproducibility between samples.

#### 2.3.3 KEGG enrichment analysis of differentially expressed genes

We conducted the analysis of differentially expressed genes (DEGs) using the DESeq2 R package. Genes with an adjusted *p*-value of less than 0.05 and a fold change greater than 2 or less than 0.5 were defined as DEGs. The genes were mapped using the Org. Hs.e.g.,.db database, and KEGG enrichment analysis was performed using the enrich KEGG function from the clusterProfiler package (Yu et al., 2012). Pathways with an adjusted *P* < 0.05 were considered significantly enriched.

#### 2.3.4 Gene set enrichment analysis (GSEA)

Gene Set Enrichment Analysis (GSEA) was performed using ClusterProfiler. Pathway-related genes were ranked based on common pathway gene sets between the experimental and control groups. The enriched gene sets were ranked according to their enrichment scores and significance levels, followed by corrections for multiple hypothesis testing. The enrichment results were visualized using the GseaVis software package.

### 2.4 Animal experiments

#### 2.4.1 Experimental animals

Six–eight-week-old C57BL/6 male mice were purchased from Rise Mice Biotechnology Co., Ltd. (Zhaoqing, China) under Permission No SCXK YUE 2020-0053. The study was conducted at the Laboratory Animal Center of the International Institute for Translational Chinese Medicine, Guangzhou University of Chinese Medicine (License No SYXK YUE 2024-0144). Their food and water were pathogen-free; the mice were maintained in a 12-h light-dark cycle at 70% humidity and 22°C. The Ethics Committee of the International Institute for Translational Chinese Medicine approved all animal studies and ensured compliance with the Laboratory Animal Care and Use Guidelines (ACU170904).

#### 2.4.2 UC induced with DSS

The mice were divided into five groups: a control group (distilled water), a model group (3% DSS), a DSS with mesalazine group (600 mg/kg), and two DSS with quercetin groups (5 and 10 mg/kg). Except for the control group, all mice received 3% DSS in their drinking water for 7 days, followed by distilled water for 4 days. All the mice underwent daily intragastric administration for 11 consecutive days. The control and model groups were intragastrically administered distilled water, whereas the DSS and mesalamine groups, as well as the quercetin group, received their respective drugs via intragastric administration.

### 2.5 Evaluation of disease activity index (DAI)

Analysis of the efficacy of drug treatment for UC in mice involved the establishment of a UC model, followed by medication therapy, with the health of the mice monitored daily. Following the guidelines of earlier research, the fecal characteristics of the mice and the presence of occult blood in the stool or bloody stools were closely observed. Once the model was established and medication treatment was completed, the mice were euthanized, and their colon lengths was measured.

### 2.6 Effect of quercetin on hemorheology in UC mice

TNF-α and IL-1β levels were measured using ELISA. Whole blood from dissected mice was centrifuged to obtain serum, which was then further centrifuged (4°C, 1,000× g, 15 min). The supernatant was analyzed using ELISA kits for TNF-α (JL10484) and IL-1β (JL18442) from JONLN, China, according to the instructions.

### 2.7 Haematoxylin and eosin (H&E) and Alcian blue staining

After the colon was removed, the luminal contents were washed with cold 1 × PBS. The tissue was then immersed in 4% paraformaldehyde for 24 h, dehydrated with gradient ethyl alcohol, embedded in paraffin, and sectioned into 4 µm slices. Histological staining was performed using hematoxylin and eosin (H&E) as well as Alcian blue staining, and the scores were assessed as previously described (Wu et al., 2020).

### 2.8 Expression of CXCL8/CXCR1/2 proteins in the colonic tissues of UC mice by immunohistochemical method

CXCL8 and CXCR1/2 were diluted with 3% BSA and incubated at 4°C overnight after deparaffinizing, thermal repairing, blocking, and closing paraffin sections. (CXCL8, Proteintech, diluted 1:400; CXCR1, Affinity, diluted 1:400; CXCR2, Proteintech, diluted 1:400, incubated). Following the incubation with the secondary antibody, color was developed with DAB, and nuclei were re-stained with hematoxylin the following day. After sealing and drying, the images were photographed microscopically, and the relative expression of CXCL8-CXCR1/2 proteins in each image was determined using an image analysis system.

### 2.9 Western blotting of proteins

Colon proteins from mice were extracted using RIPA lysis buffer with a protease inhibitor cocktail at 4°C. The samples were then separated by SDS-PAGE, transferred to a PVDF membrane, blocked with 5% BSA, and incubated with a primary antibody at 4°C for 14 h (CXCL8, Proteintech, diluted 1:1,000; CXCR1, Affinity, diluted 1:1,000; CXCR2, Proteintech, diluted 1:1,000; IL-17A, BOSTER, diluted 1:1,000; NF-κB, Affinity, diluted 1:1,000; ZO-1, Affinity, diluted 1:2,000; MUC2, Abcam, diluted 1:2,000; Occludin, Affinity, diluted 1:1,000; GAPDH, Servicebio, diluted 1:4,000). After four washes with 5% 1 × TBST, the PVDF membrane was incubated with secondary horseradish peroxidase (HRP) antibodies and detected using an ECL system with an ECL illuminating solution (Affinity, United States). GAPDH was used as an endogenous control. Finally, ImageJ software was used to evaluate the data.

### 2.10 Reverse transcription and qRT-PCR

We extracted 500 ng of total RNA using an RNA-Quick Purification Kit (Yeasen, RN001) and synthesized cDNA using a Fast All-in-One RT Kit (Yeasen, RT001). For qRT-PCR, we employed the 2 × Super SYBR Green qPCR master mix (Yeasen, QP002) and analyzed relative target expressions via the 2^−ΔΔCT^ method, using GAPDH as the control.

### 2.11 Statistical analysis

Statistical analysis was conducted using SPSS 26.0. Independent samples between two groups were compared using the *t*-test, whereas comparisons among multiple groups were performed using one-way ANOVA. Results for numerical variables are presented as mean ± standard error (±SEM). Statistical significance was set at *p* < 0.05. All statistical graphs were generated using GraphPad Prism (version 8.0.1).

## 3 Results

### 3.1 Potential target of Que in UC: network pharmacology

**FIGURE 1:**
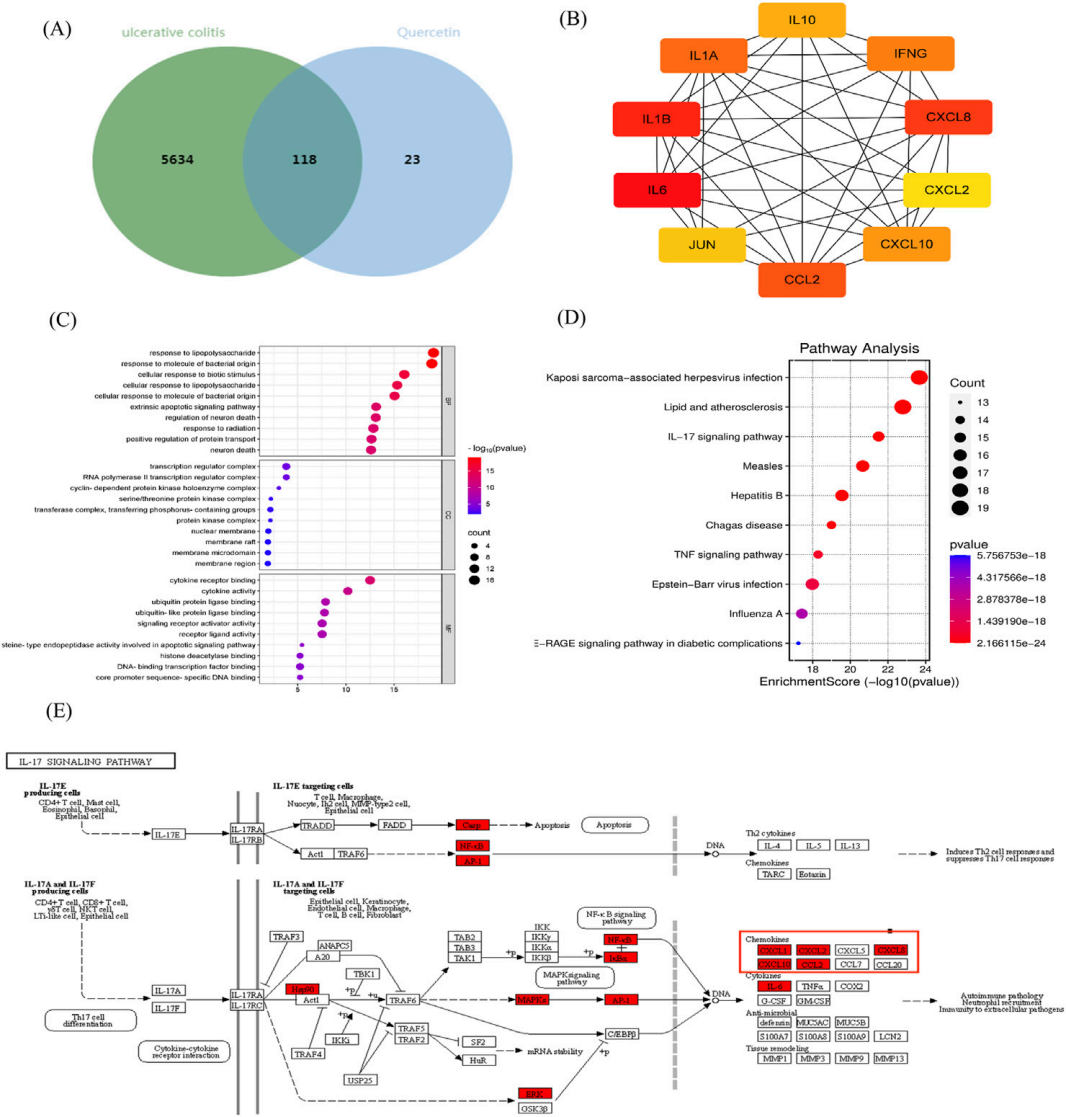
Network pharmacological analysis. **(A)** Venn diagram showing the intersection between UC and Que. **(B)** PPI interaction network of Que and UC intersection targets. **(C)** GO biofunction and **(D)** KEGG pathway enrichment analyses. **(E)** Action locations of CXCL8 target.

We identified 141 action targets for Que, including CXCL8, and 5,752 potential targets related to UC using “ulcerative colitis” as a keyword. Utilizing Venny 2.1, we found 118 common targets were identified (Figure 1A). CXCL8 was highly ranked within this network (Figure 1B). An in-depth analysis of the GO enrichment data revealed that 2,107 biofunctional entries (biological processes, cellular components, and molecular functions) were enriched in biological processes. KEGG pathway enrichment analysis identified 181 signaling pathways, with the top-ranked entries selected for visualization (Figures 1C, D). Additionally, we located the CXCL8 target within the IL-17 signaling pathway (Figure 1E). IL-17, produced by Th17 and innate immune cells, significantly contributed to inflammatory and autoimmune diseases by activating pathways such as NF-κB through its receptor. Upon activation by factors like bacterial infections, NF-κB translocates to the nucleus, binds to the CXCL8 gene promoter, and enhances its transcription. This pathway activation promotes CXCL8 transcription, thereby amplifying its role in inflammation. Consequently, we propose that CXCL8 plays a critical role in UC because of due to its involvement in the inflammatory process and its regulatory effects on the movement and activation of immune cells. Inhibition of the CXCL8-CXCR1/2 signaling pathway can significantly mitigate the symptoms of inflammatory diseases and facilitate the repair of damaged tissues.

### 3.2 CXCL8-CXCR1/2 protein expression in human colon samples

**FIGURE 2:**
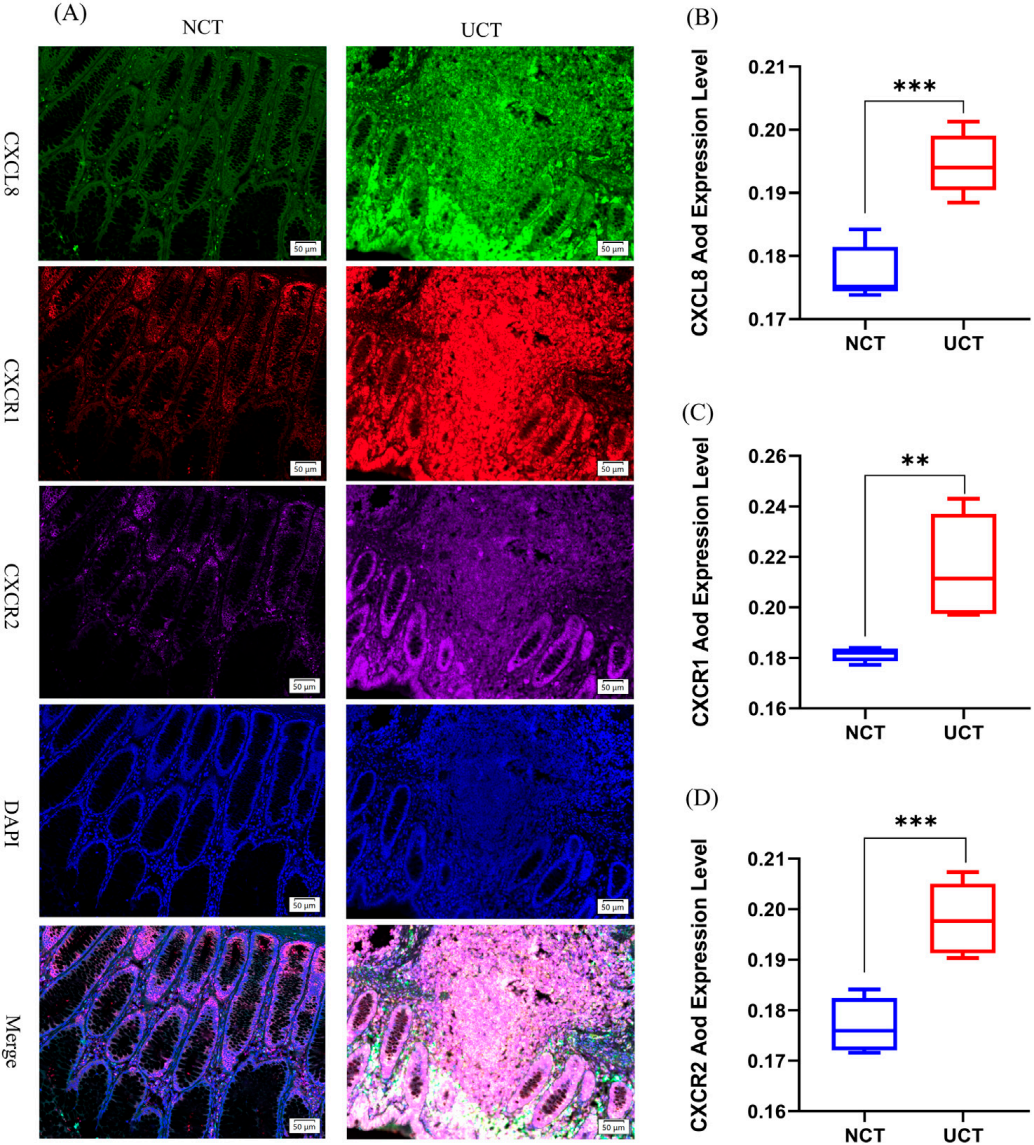
Expression changes of CXCL8-CXCR1/2 in UC tissue versus normal colon tissue. **(A)** Multiplex fluorescent immunohistochemistry was used to measure CXCL8, CXCR1, and CXCR2 protein levels in normal colon tissue (NCT) and ulcerative colitis tissues (UCT). CXCL8 was labeled in green, CXCR1 was labeled in red, CXCR2 was labeled purple, and DAPI was used to label nuclei in blue (magnification ×200); **(B–D)** Changes in AOD (average optical density) protein expression levels of CXCL8-CXCR1/2 in different intestinal tissues (n = 5). *p < 0.05, **p < 0.01, ***p < 0.001 vs. NCT.

To validate this hypothesis, we conducted multiple immunofluorescence experiments on tissue sections from patients with UC to detect relevant proteins. Our study focused on elucidating the differences between pathological tissues from patients with UC and normal colon tissues. First, we selected tissue sections from both patients with acute-phase UC and those with normal colon tissues and performed quadruple immunofluorescence staining of intestinal tissue samples using CXCL8-CXCR1/2 antibodies. Specifically, CXCL8 was labeled green, CXCR1 red, CXCR2 purple, and DAPI (nuclear stain) blue (Figure 2A). CXCL8 was expressed in both UC tissues (UCT) and normal colon tissues (NCTs), with the most pronounced expression observed in patients with acute-phase UC, whereas it was barely expressed in NCTs. Notably, CXCL8 was present in the cytoplasm of intestinal mucosal glandular epithelial cells, the nuclei of surrounding cells, and the intercellular matrix. Quantitative analysis using ImageJ showed a significant trend in CXCL8 expression, with UCA > NCT (*p* < 0.001; Figure 2B), indicating a notable difference between the groups.

Second, CXCR1 was expressed in both UCA and NCT, with the most distinct expression observed in patients with acute-phase UC. The expression trend was as follows: UCT > NCT (*p* < 0.01; Figure 2C), showing a statistically significant difference. Glandular epithelial cells of the intestinal mucosa expressed CXCR1 in the nuclei and interstices.

Additionally, although CXCR2 was expressed to varying degrees across different stages, its expression was weaker than that of CXCR1 and CXCL8 in patients with acute-phase UC. Notably, the expression trend of CXCR2 mirrored that of the former two, with UCT > NCT (*p* < 0.001; Figure 2D), demonstrating a statistically significant difference between the two groups. CXCR2 was expressed in similar locations. The CXCL8-CXCR1/2 pathway is significantly expressed during the acute phase of UC, which corroborates our previous findings. CXCL8, CXCR1, and CXCR2 have the potential to serve as predictive gene targets in patients with UC.

### 3.3 Transcriptome analysis

**FIGURE 3:**
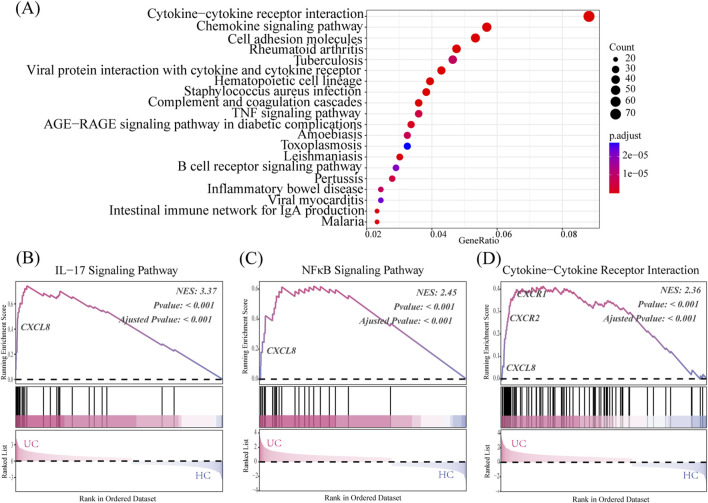
CXCL8-CXCR1/2 gene expression in the transcriptome analysis. **(A)** KEGG pathway enrichment reveals the UC’s top 20 significantly enriched pathways group. **(B–D)** GSEA results compare the UC group to the HC group, using the Normalized Enrichment Score (NES) as a key metric. NES >0 indicates core genes on the left of the peak, while NES <0 indicates core genes on the right. Significance of the Enrichment Score (ES) is assessed using the *p*-value.

The KEGG pathway enrichment results showed that the UC group had upregulated pathways compared to the healthy control (HC) group, mainly involving cytokine–cytokine receptor interactions, chemokine signaling pathways, and TNF signaling pathways (Figure 3A).

As shown in Figures 3B–D, the UC group exhibits significant enrichment in IL-17 signaling, NF-κB signaling, and cytokine–cytokine receptor interaction genes compared to the HC group (*p* < 0.05). Notably, CXCL8 serves as a core gene for all three pathways and shows significant differences in expression when comparing the UC and HC groups. CXCR1 and CXCR2 are key genes in the cytokine–cytokine receptor interaction pathway, showing significant overexpression in the UC group compared to the HC group, which affects the pathway’s differential expression.

### 3.4 Que in acute enteritis

**FIGURE 4:**
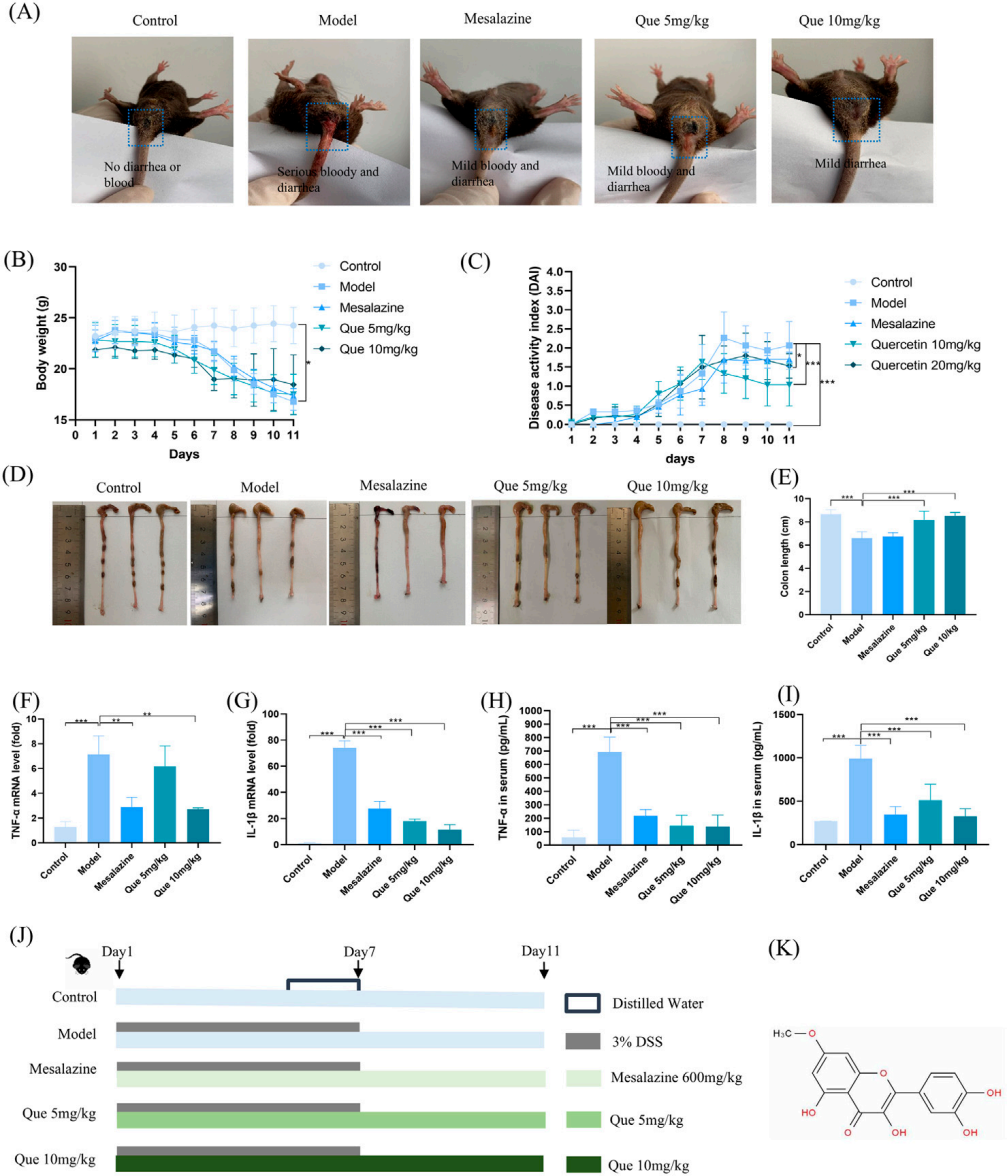
Que mitigated DSS-induced ulcerative colitis. **(A)** Perianal conditions of mice in each group. **(B)** Body weight changes in mice (n = 10). **(C)** Variations in the disease activity index (DAI) in mice (n = 10). **(D–E)** Colon length measurements in mice (n = 10). **(F–G)** mRNA expression levels of TNF-α and IL-1β in the colon as determined by qPCR (n = 3). **(H, I)** Serum levels of TNF-α and IL-1β (n = 6). **(J)** Experimental design. **(K)** Chemical structure of Que. *p < 0.05, **p < 0.01, ***p < 0.001.

The perianal conditions of mice on day seven of the experiment are shown in Figure 4A. The control group did not exhibit hematochezia, whereas the model group exhibited severe hematochezia. In the mesalazine and Que groups, the severity of blood loss in the stool was mitigated, and the degree of alleviation positively correlated with the Que dose, suggesting that Que has a therapeutic effect on acute enteritis. In addition, the experimental group exhibited a significant weight reduction after day five compared with that in the control group (Figure 4B). Mesalazine at a dose of 600 mg/kg also mitigated the reduction in body weight compared to the model group. The disease activity index (DAI) of the model group was significantly elevated relative to the control group. Following the administration of Que at 5 mg/kg and 10 mg/kg, the DAI decreased significantly, with the observed differences being statistically significant (Figure 4C). Morphological examination of the colon revealed that the colon in the model group exhibited a significant reduction in length, accompanied by the development of hyperemia and edema. Treatment with mesalazine and Que resulted in marked improvements in both the morphology and length of the colon in UC mice, with Que at a dose of 10 mg/kg demonstrating pronounced effects (Figures 4D, E).

Storms of inflammatory cytokines are common during colitis, and changes in cytokine levels may affect the rapid progression of inflammatory colitis. As UC progresses, the cytokine levels reflect the inflammatory status of the body. Mice in the model group had significant increases in TNF-α and IL-1β levels compared to control group mice in the ELISA results (Figures 4H, I). This upregulation was consistent with the observed mRNA levels of TNF-α and IL-1β in the colon tissue (Figures 4F, G). TNF-α and IL-1β levels in colon tissue and serum were markedly reduced by Que treatment. In Figures 4J, K, we can see the experiment design and the chemical structure of Quercetin.

### 3.5 Que improved the intestinal epithelial barrier of UC mice

**FIGURE 5:**
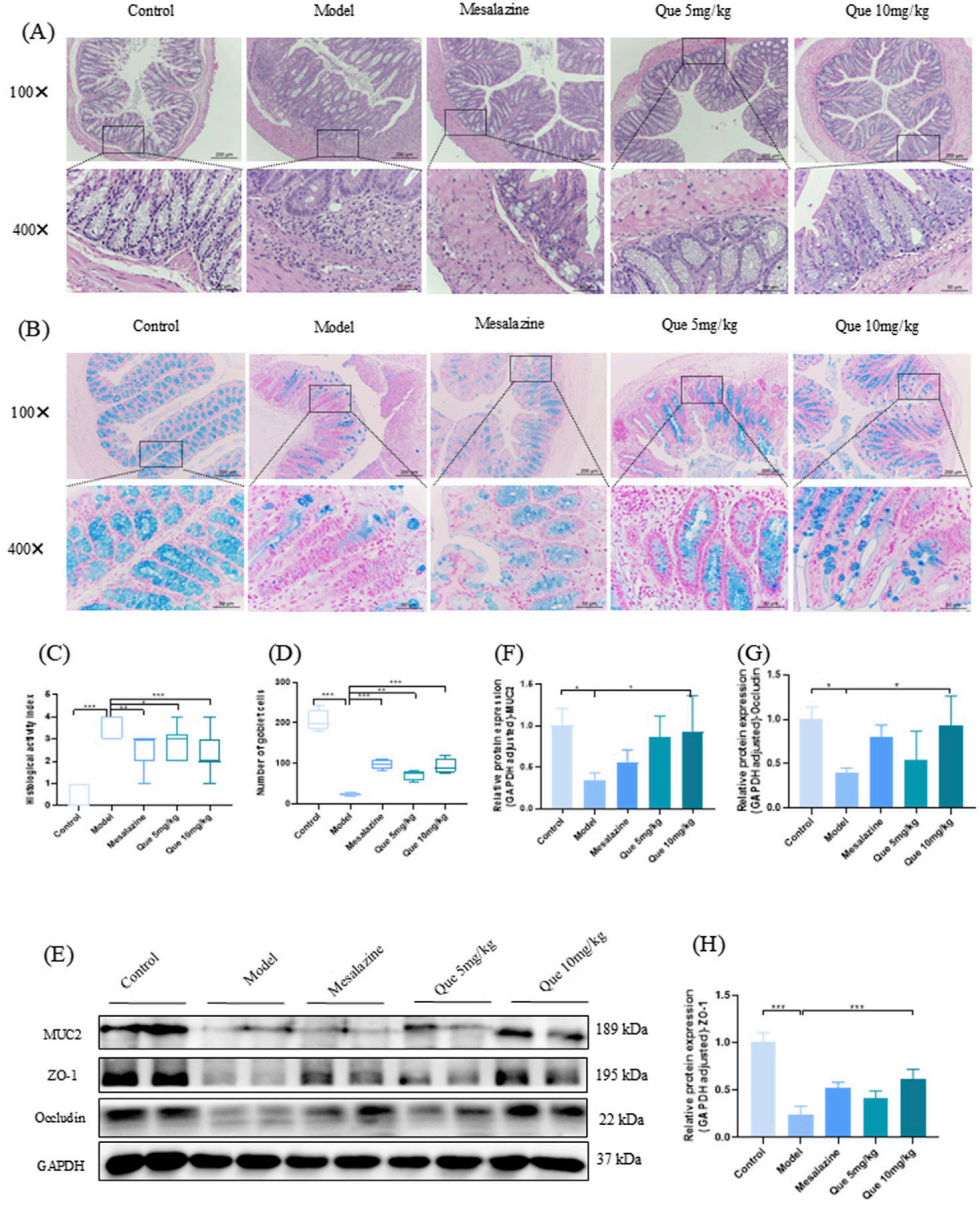
Que restores the integrity of the intestinal mucosal barrier in DSS-induced murine models. **(A, C)** Hematoxylin and eosin staining of colon tissue sections (×100 and ×400 magnification, respectively) and histopathological scores (n = 4). **(B, D)** Alcian blue staining (×100 and ×400) of goblet cells in mouse colons. (n = 4). **(E–H)** MUC2, ZO-1, and Occludin levels in mouse colons (n = 4). *p < 0.05, **p < 0.01, ***p < 0.001.

The control group displayed well-organized intestinal glands and preserved structural integrity upon H&E staining of the colon. In contrast, the model group exhibited pronounced ulceration, disorganized and loosely arranged intestinal glands, significant infiltration of inflammatory cells, localized hyperemia, and edema. Notably, treatment with mesalazine and Que substantially restored colonic structural integrity, with marked reductions in ulceration and inflammatory cell infiltration (Figures 5A, B). Significant increases in goblet cells within colonic tissues were observed in Que-treated mice (Figures 5C, D). Additionally, the levels of tight junction (TJ) proteins ZO-1, MUC2, and occludin were elevated in the treated mice (Figures 5E–H). Overall, Que restored intestinal barrier integrity in compromised animals.

### 3.6 Que activates CXCL8-CXCR1/2 axis in DSS-induced mice

**FIGURE 6:**
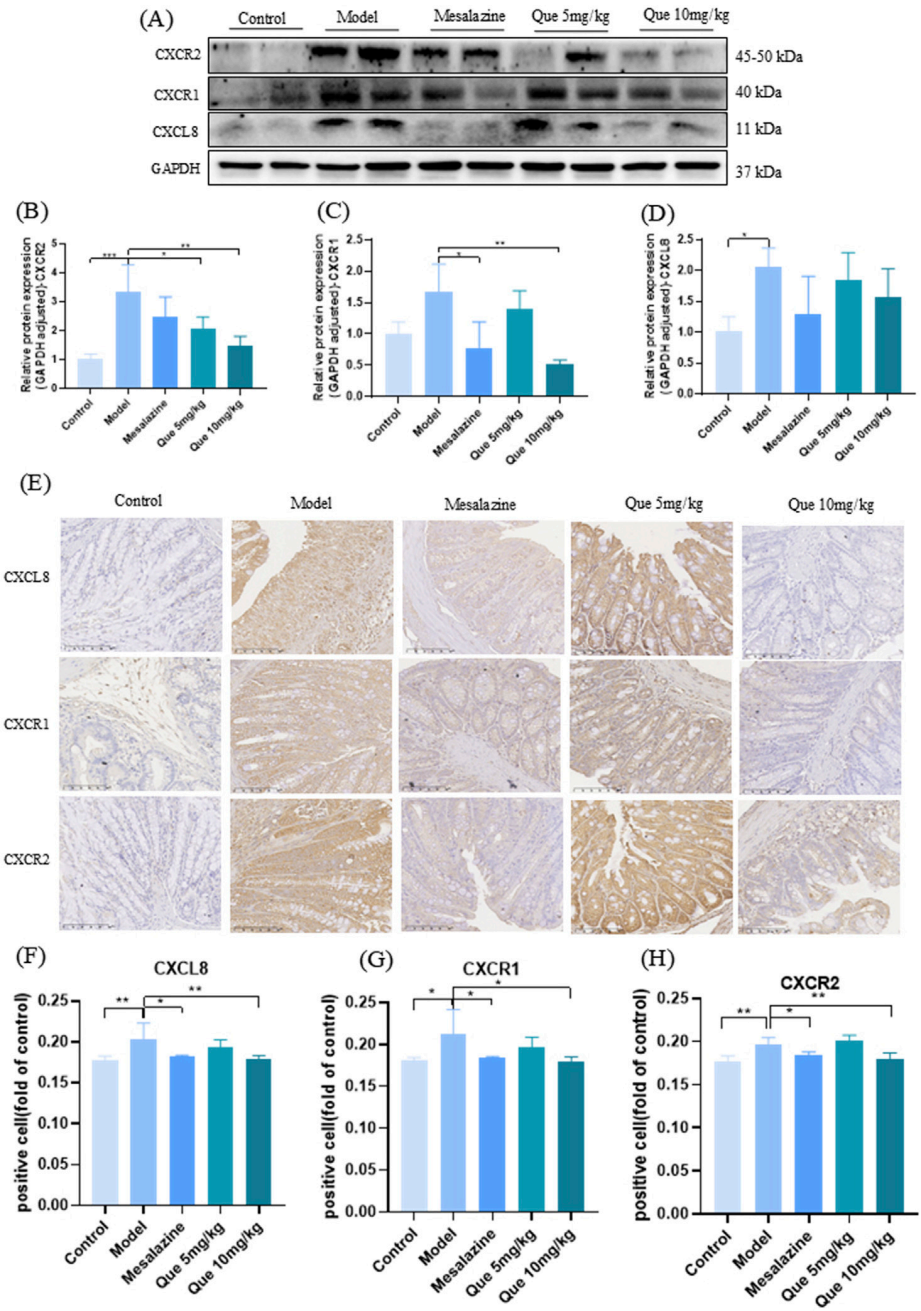
Que regulated the CXCL8-CXCR1/2 axis. **(A–D)** CXCL8, CXCR1, and CXCR2 expression levels in mice’s colonic tissue (n = 4). **(E–H)** IHC expression of CXCL8, CXCR1, and CXCR2 in the colon (×100, ×400) (n = 4).*p < 0.05, **p < 0.01, ***p < 0.001.

Figures 6A–D shows that Que treatment reduced CXCL8, CXCR1, and CXCR2 levels in a dose-dependent manner compared to the model group (*p* < 0.05). However, Que combined with mesalazine increased these protein levels compared to those in the control group (*p* < 0.05), as confirmed by IHC analyses (Figures 6E–H). These results suggest that Que may alleviate UC by modulating the CXCL8-CXCR1/2 axis.

### 3.7 Effect of Que on IL-17 signaling

**FIGURE 7:**
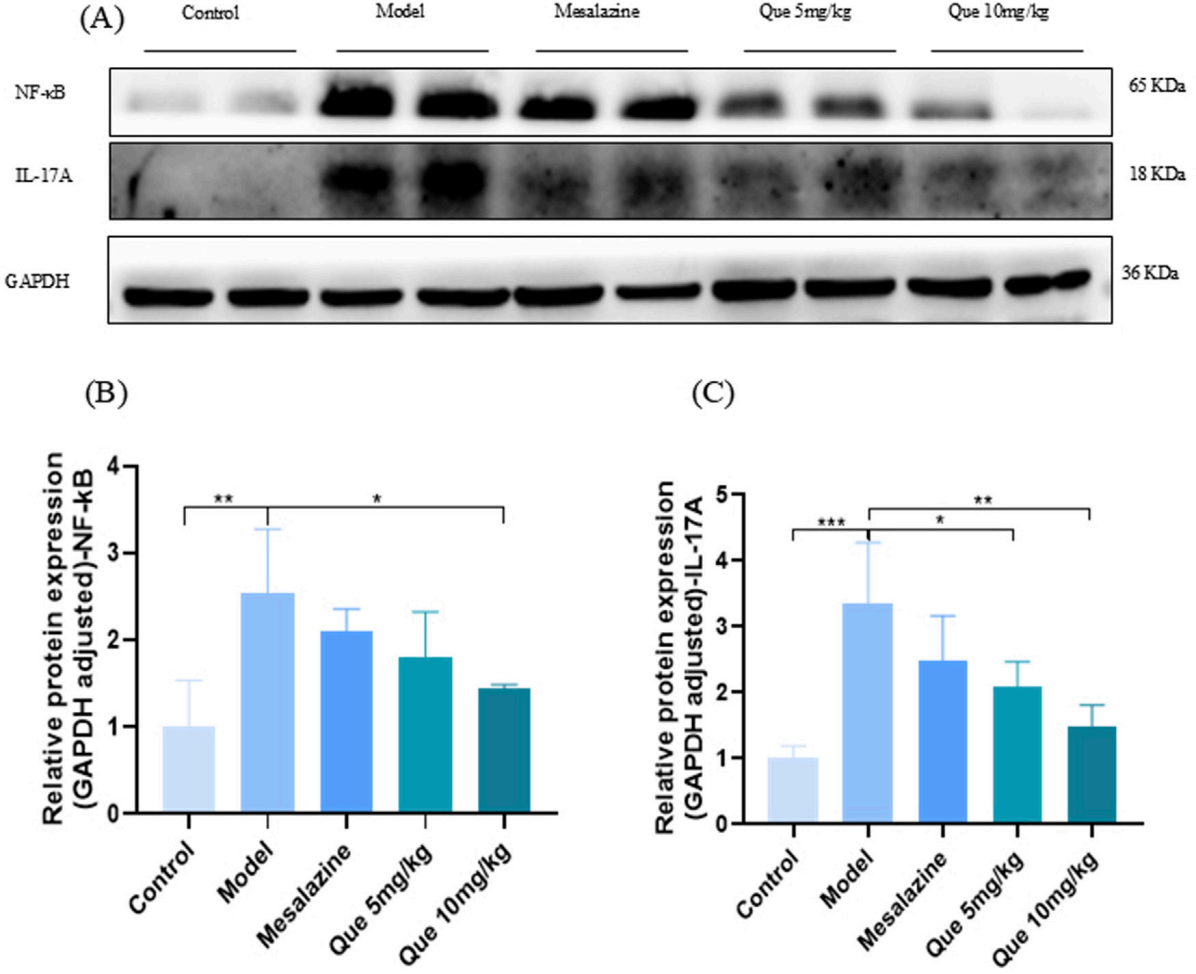
Que decreases the protein expression level of IL-17A and NF-κB. **(A–C)** Protein expression of IL-17A and NF-κB in colon of mice (n = 4). *p < 0.05, **p < 0.01, ***p < 0.001.

As illustrated in Figures 7A–C, Que administration significantly decreased the expression of IL-17A and NF-κB proteins in a dose-dependent manner relative to the model group (*p* < 0.05). Conversely, the combined treatment of Que and mesalazine resulted in an increase in the protein levels of IL-17A and NF-κB when compared to the control group (*p* < 0.05). These findings indicate that Que may alleviate UC by modulating the IL-17 signaling pathway.

## 4 Discussion

The pathogenesis of UC is unclear; however, it is characterized by damage to the intestinal barrier. Therefore, effective and novel therapeutic agents are urgently needed. Our study found that Que significantly improved weight loss, diarrhea, and bloody stools in DSS-induced UC mice while aiding in epithelial cell repair and restoring intestinal barrier function. Furthermore, we provided evidence that Que can inhibit IL-17A signaling and its downstream inflammatory pathways, such as the CXCL8-CXCR1/2 signaling axis, thereby mitigating UC and modulating both inflammation and intestinal barrier function *in vivo*. In conclusion, our findings indicated that Que may serve as a promising novel therapeutic agent for UC. The *in vivo* modulation of inflammation and intestinal barrier function highlights its potential as a treatment for UC.

Potential therapeutic targets for the treatment of UC were identified through the PPI network, highlighting CXCL8, CXCR1, CXCR2, IL17A, and IL1β. To enhance the reliability of the results of the network pharmacological analysis, colon and normal tissues from patients with UC were collected and subjected to multiple fluorescence immunohistochemical staining assays. These assays revealed that increased expression of the CXCL8-CXCR1/2 proteins is a significant characteristic of UC pathology. Notably, this finding was further corroborated by transcriptome analysis. Furthermore, the analysis showed that CXCL8, a pivotal gene in both the IL-17 and NF-κB signaling pathways, plays a significant role in the differential expression observed between UC and HC. Previous studies have demonstrated a strong association between the CXCL8-CXCR1/2 axis and processes, such as inflammation, tumorigenesis, invasion, and metastasis (Mishra et al., 2021). Consequently, CXCL8, along with its receptor derivatives and inhibitors, haves emerged as a prominent focus of clinical research. Inhibiting the CXCL8-CXCR1/2 axis not only alleviates inflammation but also suppresses tumor cell proliferation, thereby impeding disease progression (Sitaru et al., 2023b). Research indicates that the CXCL8-CXCR1/2 axis may play a role in the immune response to invading pathogens by downregulating the expression of CXCL8, CXCR1, and CXCR2, which in turn suppresses the inflammatory response (Han et al., 2021). Consequently, our findings suggest that the CXCL8-CXCR1/2 signaling axis may serve as a promising target for immunotherapeutic interventions.

The DSS-induced UC model effectively replicated the characteristic symptoms and pathological alterations observed in patients with UC, including weight loss, diarrhea, bloody stools, extensive colonic inflammation, and intestinal barrier disruption. This model has emerged as a crucial instrument for elucidating the pathogenesis of UC and evaluating novel therapeutic agents for its treatment (Huangfu et al., 2020). Quercetin, a natural flavonoid polyphenol, has emerged as a significant tool in the exploration of UC pathogenesis and the investigation of new therapeutic agents for its treatment. Due to its anti-inflammatory, antimicrobial, and antitumor properties, quercetin is extensively used in the clinical management of various diseases, including cardiovascular disorders and malignancies. Its potential as a natural compound for comprehensive therapeutic applications is noteworthy (Hosseini et al., 2021; Singh et al., 2021). Prior research has established that quercetin exerts notable anti-inflammatory effects by inhibiting the activation of the nuclear transcription factor NF-κB and diminishing the release of pro-inflammatory cytokines while engaging in other anti-inflammatory pathways to safeguard against cellular damage. Nevertheless, the potential involvement of quercetin in the therapeutic management of inflammation-induced intestinal disorders, such as UC, via the CXCL8-CXCR1/2 signaling pathway, remains uncertain. To explore the protective efficacy of quercetin against UC, a mouse model of DSS-induced UC was used. Our results demonstrated that quercetin effectively treated UC mice by reducing weight loss, diarrhea, blood in the stool, and colonic injury.

Inflammation and intestinal barrier dysfunction play important roles in the pathogenesis and progression of UC and are key to understanding its pathogenesis as well as exploring drugs to prevent and treat UC (Nalleweg et al., 2015). The intestinal epithelium serves as the largest interface between an organism and the external environment, forming a crucial barrier against external threats. TJs consist of a macromolecular complex of transmembrane proteins and cytoplasmic proteins, primarily functioning as a ‘barrier’ and ‘fence’’ that relies on transmembrane proteins (e.g., occludin) and scaffolding proteins to protect the intestinal epithelium. Occludin and junctional proteins (e.g., ZO-1) regulate the function of the gate, maintaining homeostasis of the intestinal barrier and regulating intestinal permeability (Kuo et al., 2022; Pan et al., 2023). The secretion of MUC2 by goblet cells renews the intestinal mucus layer and contributes to the formation of the intestinal mucosal barrier. When the intestinal mucus layer is damaged and thinned, intestinal bacteria can cross the mucus layer to reach and affect the intestinal epithelial cells. In response, goblet cells can secrete large amounts of MUC2 in a compensatory manner, which helps flush bacteria from the surface of intestinal epithelial cells, repair the intestinal mucus layer, and maintain the balance of the intestinal l environment (Luis and Hansson, 2023). Knockdown of ZO-1, occludin, or MUC2 in mice impairs intestinal mucosal repair and increases the risk of intestinal inflammation (Kuo et al., 2021; Van der Sluis et al., 2006). Notably, this study demonstrated that Que effectively mitigated the DSS-induced enhancement of intestinal permeability and compromised intestinal barrier integrity. These results suggest that the protective effect of Que against UC may be closely related to the suppression of the inflammatory response and the maintenance of intestinal barrier integrity.

**FIGURE 8:**
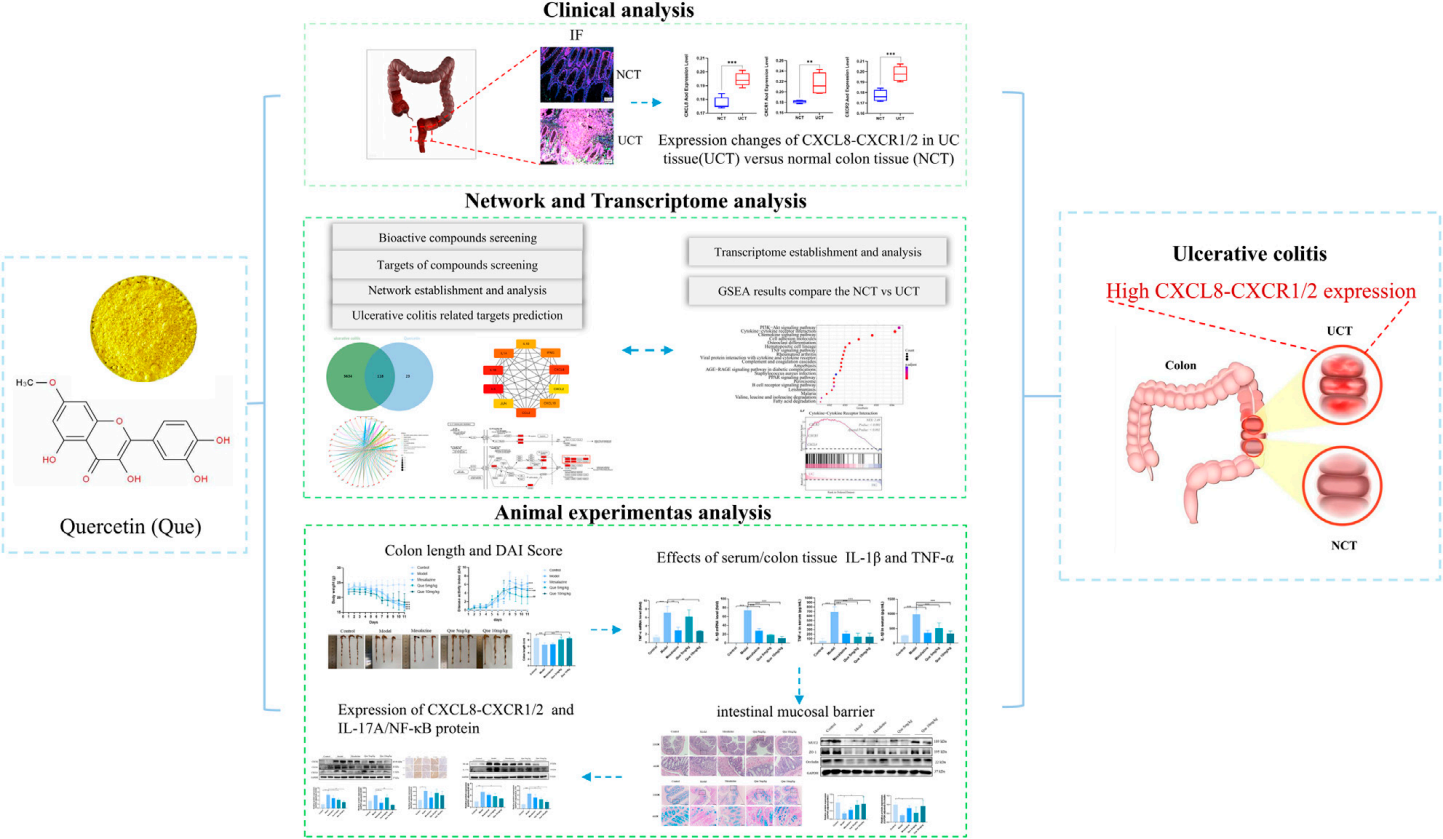
Workflow for analyzing Que’s mechanisms in UC treatment. Network pharmacology, in conjunction with transcriptomic methodologies and experimental validation, demonstrated that quercetin alleviated the symptoms associated with DSS-induced colitis. It attenuates the inflammatory response, promotes the repair of colonic mucosal tissues, and elevates the levels of colonic tight junction proteins in mice with ulcerative colitis. These effects may enhance tight junction integrity and restore intestinal barrier function by modulating the IL-17A signaling pathway while simultaneously inhibiting the CXCL8-CXCR1/2 axis.

KEGG pathway enrichment analysis showed that Que’s intervention in UC mainly involves the IL-17 signaling pathway, TNF signaling pathway, and others, with the IL-17 signaling pathway being closely related to the development of UC. IL-17 (also known as IL-17A), the earliest discovered and most widely studied pro-inflammatory cytokine of the IL-17 family, is expressed in large quantities in the intestinal mucosa of patients with UC, inducing the release of various pro-inflammatory cytokines and chemokines (Zhang et al., 2018). Previous studies have demonstrated that IL-17 is heavily upregulated in the intestinal mucosa of patients with IBD, and its pathological production leads to excessive inflammation and significant tissue damage (Amatya et al., 2017). In addition, during the pathogenesis of UC, inflammatory factors such as IL-6, TNF-α, and IL-1β are hyper-expressed, leading to a more severe inflammatory response and tissue damage in intestinal tissues while disrupting the intestinal mucosal environment (Chung et al., 2017). Our results suggest that Que may inhibit the expression of downstream CXCL8 and its receptor proteins CXCR1 and CXCR2 by suppressing the expression levels of IL-17 and NF-κB; and by decreasing the release of pro-inflammatory factors TNF-α and IL-1β, thus exerting anti-inflammatory effects and reducing pathological injury in the colon of UC-model mice, which verifies the relationship between cyberpharmacology and transcriptomic methodology (Figure 8).

In conclusion, the current findings demonstrate that quercetin can mitigate DSS-induced colitis in mice by reducing the inflammatory response and preserving the stability of the intestinal mucosal barrier, thereby offering novel evidence. Furthermore, Que directly decreased the protein expression levels of CXCL8, CXCR1, CXCR2, IL-17A, and NF-κB, contributing to its protective effect against UC. These results indicated that quercetin is a promising therapeutic agent for UC.

Similar to most research endeavors, the design of the present study was constrained by certain limitations. First, in this study we applied network pharmacology to identify quercetin as the principal therapeutic target for UC. Nonetheless, the efficacy of network pharmacology is contingent on the availability of extensive biological data and compound information, and the quality and comprehensiveness of these datasets directly influence the reliability of the findings. Databases may contain errors, inconsistencies, or incomplete data. Consequently, follow-up experiments were performed to ascertain whether quercetin exerts therapeutic effects on UC via these specific targets. These findings confirm the viability of employing network pharmacology for screening compounds and targets. Future research should integrate biosequencing with network pharmacology post-administration for UC to enhance the scientific rigor of targeted screening. Furthermore, this study focused on CXCL8-CXCR1/2 protein expression but did not examine the downstream mechanisms of CXCL8-CXCR1/2. Additionally, we did not explore how the drug degraded after binding to the target; this will be further investigated in future *in vitro* cellular experiments or through the knockdown of related genes.

## Data Availability

The original contributions presented in the study are included in the article/supplementary material, further inquiries can be directed to the corresponding authors.
